# Co-design rationales as living documents for reporting the exchange of experiential, professional and scientific perspectives in mental health care innovation

**DOI:** 10.1371/journal.pmen.0000689

**Published:** 2026-08-20

**Authors:** Lars Veldmeijer, Bard Wartena, Gijs Terlouw

**Affiliations:** 1 NHL Stenden University of Applied Sciences, Research Group Health Innovation, Leeuwarden, The Netherlands; 2 UMC Utrecht, Division Neuroscience, Utrecht, The Netherlands; PLOS: Public Library of Science, UNITED KINGDOM OF GREAT BRITAIN AND NORTHERN IRELAND

## Abstract

Co-design has become a prominent approach in mental health care innovation, reflecting an increasing recognition of epistemic pluralism as much-needed for participatory change. However, despite its growing adoption, co-design studies frequently offer limited insight into how these exact collaborations influenced the resulting artefacts or change. Drawing on over a decade of co-design practice in mental health care, this essay identifies lessons related to epistemic pluralism, dissensus, serendipity, contextual constraints, participatory approaches, paradigmatic differences, and power relations. We make the argument that these challenges arise from a broader reporting gap: the lack of systematic accounts detailing how various ways of knowing contribute throughout the co-design process. To address this issue, the concept of co-design rationales is introduced, defined as a framework for documenting the development of design decisions through co-learning among stakeholders with experiential, professional, and scientific expertise. Building on established design rationale literature, we develop an architecture that is operationalized through a co-design rationale canvas, enabling the iterative documentation of both the design process and the collaborative production of knowledge. Co-design rationales, as living documents, updated across iterations, discussed with stakeholders, and used to support shared decision-making, clarify who contributed, how decisions were negotiated, which forms of knowledge informed design choices, and how these choices shaped the final artefact or change. Beyond this, we postulate that reciprocity serves as the relational condition, or hallmark, of co-design, enabling epistemic pluralism and collective learning. Without co-design rationales, we may know that stakeholders participated, but not how their ways of knowing shaped the outcome or where reciprocity broke down.

## Introduction

Design is a situated and local endeavour that involves diverse stakeholders [1]. Unlike traditional sciences, which seek to explain the natural world, design aims to shape the artificial world [2]. Designers seek to understand the world through the development of frames, as framing and reframing help to perceive challenges from new perspectives and to explore potential futures [3]. As a result, design knowledge pertains to the human-made world and the ways in which individuals can contribute to its creation and maintenance [2]. At the same time, design outcomes and processes also serve as an encapsulation – expressing or showing the most important facts about the design outcome – of situated, provisional, and generative knowledge [4–6].

Over the years, design research has matured and entered a new era [7], and the uptake of design approaches across disciplines and fields has exploded [8]. Within design research, co-design, as a collaborative approach to design practice and research, has established a significant methodological role in participatory mental health research [9–11]. Literature reviews indicate that co-design is defined in numerous ways [12,13] and that the description and application of co-design methodology vary considerably depending on the adopted definition and the phase of the process in which it is implemented [14]. Another co-design review indicates that participant roles range from informants to co-designers, while numerous studies do not progress to prototype development or testing [13]. Co-design frequently involves individuals who are not professional designers, engaging them to develop innovative ideas informed by their experiences, values, visions, and collaborative practices [15]. Despite definitional differences, all interpretations agree that co-design is a collective process rather than a simple act of consultation. Within the broader methodological continuum of participatory design, co-design in mental health care seeks to bridge research and practice by involving diverse stakeholders as experts of their own experiences.

In the present essay, we follow Sanders and Stappers’ [16] use of the term co-design to indicate “collective creativity as it is applied across the whole span of a design process.” Although the terms co-design, co-production, and co-creation are often used interchangeably, they denote distinct forms of collaboration. According to Vargas et al. [17], co-design refers to the collaborative development of design solutions in response to a prespecified problem. In contrast, co-production engages stakeholders in executing a previously agreed solution to an established problem and emphasizes the allocation of resources and assets within these constraints to achieve improved outcomes. Co-creation serves as an overarching concept that encompasses various forms of collaborative value creation. In this essay, we specifically focus on co-design, with particular attention to how collaborative design reasoning influences design decisions and the resulting artefact or purposeful change [18]. Throughout this essay, the terms design artefact, design outcome, and purposeful change refer to the potential outputs of a co-design process. These may include tangible products, services, interventions, methods, or other intentional changes developed through collaborative design. Established fundamental co-design principles are inclusive, participative, equal, applicable, iterative, cooperative, and transparent [19].

The rapid expansion of co-design in mental health care is driven by several intersecting developments. A primary factor is the growing emphasis on involving service users and individuals with lived experience in shaping care and innovation processes. Experiential knowledge is increasingly recognized as important in mental health care [20], for example as a prerequisite for tailoring care and innovations to the needs of individuals facing challenging circumstances [9,21]. However, the conceptualization of experiential knowledge remains a topic of ongoing debate [22–24]. Borkman [25] provides a sociological account of experiential knowledge, defining it as “truth learned from personal experience with a phenomenon rather than truth acquired by discursive reasoning, observation, or reflection on information provided by others.” We will adopt this definition for pragmatic reasons, since consensus on this concept may never be realized. According to this definition experiential knowledge gives access to everyday aspects of illness and recovery that may remain inaccessible to professionals and researchers without lived experience of a specific phenomenon. Experiential knowledge is, however, not subordinate to professional knowledge, but it offers a unique epistemic perspective that complements other forms of understanding.

The inclusion of multiple forms of knowledge such as experiential and professional [25], as well as scientific knowledge (which is often viewed as another distinct way of knowing), does not automatically result in harmony or fruitful collaboration in co-design [26]. Each of those perspectives is grounded in different assumptions, values, and criteria for what constitutes valid evidence. For example, experiential perspectives (e.g., clients/patients, people with lived experience, experts by experience, and peer support workers) may privilege subjective meaning and personal narratives; professional perspectives (e.g., clinical and practical expertise in psychiatry, psychology, nursing, or social work) may emphasise feasibility for care practice and organisational constraints; while scientific perspectives (e.g., researchers and methodologists) may aim for methodological rigour and empirical evidence. In this essay, these perspectives are used to reflect different contributions to collaborative design reasoning and do not imply a hierarchy of expertise.

To make these perspectives more transparent in design processes, Wartena and Kuipers [27] introduced a framework that consists of ‘resonance’, ‘relevance’, and ‘rigour’ that help articulate the quality concern that stakeholders with different backgrounds often foreground and value, and therefore also exhibiting potential merit for better understanding co-design processes (the authors also refer to ‘rapidness’, which points more to the nature of design and not to a specific way of knowing, which we will return to in the lessons learned). Applying these conversational lenses, experiential perspectives are for instance more focused on resonance (e.g., the extent to which a design resonates with an individual’s lived experience), professional perspectives on relevance (e.g., the extent to which a design is relevant and beneficial in practice), while scientific perspectives value rigour (e.g., the extent to which a design is grounded in robust evidence). In short, this means that participants from different backgrounds may have diverse values (rigour, relevance or resonance), interpretations and reach different conclusions about which problems deserve attention and which solutions are desirable. Although experiential, professional, and scientific perspectives often align with resonance, relevance, and rigour, respectively, one should not regard these associations as rigid, or worse, as stereotypical categories. They are meant and used as pragmatic distinctions to show where tensions may occur. In co-design, all stakeholders are expected to engage thoughtfully with each of the dimensions.

Embracing these different ways of knowing is vital for epistemic pluralism in mental health care [28,29], however, co-design comes with its own conflicts [30] and misusing co-design risks unintended consequences and downstream harm [31,32], such as participatory assimilation [26]. Numerous scholars have advanced recommendations and insights to enhance the authenticity and transparency of co-design and participatory processes [33–35]. Some recent contributions include frameworks and guidelines for understanding stakeholder engagement throughout project lifecycles, as well as strategies to increase inclusivity while addressing power dynamics [36,37]. Additional proposals address the challenges of involving ‘extreme users’ in co-design [38]. Equally important is that designers and researchers critically evaluate their choice of participatory design approach, as, according to Terlouw and Veldmeijer [39], the valuable involvement of individuals with lived experience depends less on the specific approach and more on the degree of inclusion and the underlying power structures. Other reporting frameworks addressing patient and public involvement [40–42] do a great job at enhancing transparency in reporting the breadth of participation and collaboration, intervention descriptions, qualitative research, and implementation. However, they do not yet – nor do they aim to – systematically illuminate how collaborative design reasoning, epistemic authority, dissensus, and design decisions collectively shape the artefact or change. Recent umbrella review evidence similarly indicates that digital health intervention through co-design is often inconsistently conducted and described, underscoring the need for reporting approaches that detail how collaborative design reasoning shaped the resulting outcome [43].

The identified challenges and existing proposals reveal a reporting gap that this essay seeks to address: co-design studies in mental health care are increasing in popularity but rarely explain how experiential, professional, and scientific stakeholder contributions shape design reasoning and design decisions, and ultimately how the co-design process resulted in an artefact or a purposeful change. In the present essay, we will highlight seven lessons learned about co-design in mental health care and outline the development of key elements that can be integrated to form an architecture for what we term ‘co-design rationales’. A co-design rationale is defined as a living document that systematically documents the formation of design decisions over time through situated interactions among stakeholders with diverse expertise and varying degrees of authority. This includes accounts of how diverging epistemic contributions influence the final artefact, whether it is a tangible product or a purposeful change. Co-design rationales can be used as an integrated framework for reporting as well as a methodological tool used during co-design. Co-design is conceptualized as a collaborative, context-embedded activity that produces outcomes encapsulating the design choices and knowledge generated throughout the process [18].

We will postulate throughout this essay that the encapsulation of experiential, professional, and scientific knowledge, and the coherent narrative it conveys, is the value of co-design in mental health care. We will subsequently argue that ‘reciprocity’ constitutes a necessary relational condition that drives the co-design process. Our proposal for co-design rationales aims to contribute to a more critical and practice-oriented understanding of co-design that supports people with lived experience, professionals, and researchers in using it more transparently and carefully within mental health innovation processes.

### Lessons learned about co-design in mental health care

In this section we present seven lessons learned regarding co-design in mental health care, drawing on over ten years of co-design projects conducted by our Digital Innovation in Healthcare research group in the Netherlands. In these projects we collaborated with diverse groups of individuals with lived experience, professionals, and researchers. These lessons are organized into two domains: (1) the co-design process and (2) collaborative knowledge production. Although this distinction is somewhat artificial, as the co-design process serves as the vehicle for knowledge production, it clarifies how co-design processes generate both design outcomes – hereafter referred to as ‘design artefacts’, ‘design outcomes’, or ‘purposeful change’, all denoting the tangible results of the design process – and encapsulate knowledge and learning. This essay is a practice-based conceptual synthesis (i.e., not a systematic review or formal empirical analysis) informed by published studies, our own project documentation, and our long-term experience with involvement in co-design projects. Throughout these lessons, we address the various perspectives (experiential, professional, and scientific), and the conversational lenses we associated them with in the introduction (resonance, relevance, and rigour), to illuminate the tensions we encountered in the process. While presented as seven distinct lessons, these insights are closely interconnected and collectively motivate the need for co-design rationales.

## Domain 1: The co-design process

### Lesson 1: The co-design process is about productively working with differences

Co-design processes in mental health must accommodate multiple, potentially divergent perspectives, as these are crucial for achieving meaningful and sustainable innovation. The prefix “co-” in co-design signifies that diverse viewpoints are integral to a collaborative exploratory process. Sometimes, this exchange involves only two perspectives, while in other contexts, it encompasses a broader array of viewpoints. The prevailing paradigm in health sciences emphasizes theoretically generalizable concepts, or rigour (which we return to and expand on later in Lesson 5), which significantly shape the knowledge base and, by extension, professional practice in mental health. The literature, however, offers limited accounts of how experiential perspectives are elicited, primarily because it is often assumed that professionals or researchers identify these requirements [44]. Even when requirements are identified through ‘person-based’ approaches [45], it is still relatively rare for qualitative user study findings to be reported independently or valued equally with quantitative results in the health sciences [46]. Instead, there is a pronounced focus on developing and extending theoretical frameworks, such as those related to behaviour change or decision-making, to guide the design process. For this reason, the system perspective, typically upheld by professionals and researchers, tends to dominate, increasing the risk of superficial and tokenistic involvement of experiential perspectives [26].

This tendency is further reinforced by the fact that many innovation and implementation frameworks prioritize consensus [44,47–52]. The pursuit of consensus is not seldom approached from a single perspective, and occasionally from an assumed all-encompassing viewpoint, where all stakeholders are expected to be convinced of the innovation’s value from a particular standpoint. In practice, this is most often the professional or the scientific perspective, which are rooted in practical (relevance) and generalizable knowledge (rigour). Although this approach may effectively promote innovation within specific disciplines, it frequently restricts the inclusion of experiential perspectives (resonance), thereby undermining the collaborative intent of co-design [39].

Star and Griesemer [53] introduced the concept of the “boundary object”, which was originally intended to enable constructive collaboration between different sites or social systems without demanding consensus. Rejecting the assumption that consensus regarding goals, values, needs, and preferences is always required offers a helpful line of thinking for co-design in mental health. This boundary object perspective legitimizes the coexistence of multiple viewpoints for systemic awareness [54]. For professionals, an innovation may be relevant if it serves as a recognizable tool to facilitate care delivery, whereas for clients or people with lived experience, it may show resonance if it functions as a means to articulate their unique experiences and first-person perspectives [55] or to address everyday challenges [56,57]. For researchers, it is rigorous if it is backed by empirical evidence and theories. These perspectives can exist in harmony – the one does not exclude the other. At its core, participation in design and the presence of dissensus are inherently linked [18,58]. Embracing dissensus may allow epistemic pluralism in mental health care to flourish [28].

A systematic review [56] demonstrates that genuine curiosity about differing perspectives, coupled with active discussion and exchange, significantly increases the likelihood of successful adaptation and implementation of innovations, tools or change in health care contexts [59–63]. Engaging with a shared learning space that accommodates multiple perspectives promotes collective ownership of both the problem and its solutions and encourages ongoing consideration of diverging viewpoints. The exchange of dissenting perspectives is a critical component of the co-design process, especially in complex environments with diverse stakeholders, needs, and interests. When these factors are addressed through the framework of a boundary object and integrated into the design, mutual understanding is enhanced. Collaborative inquiry requires an environment conducive to change, in which all stakeholders are motivated to progress together as a team, which is imperative for design [3].

### Lesson 2: The co-design process and its outcomes are unpredictable

Researchers frequently approach projects with a specific solution or direction for change in mind, informed by prior research findings and established theories. This practice is common, as researchers are recognized as scientific experts in their fields. When seeking funding from grant providers, for example, they must submit extensive and detailed proposals that clearly outline the expected outcomes in exchange for investment. In contexts where innovation is central and the involvement of multiple stakeholders is much-needed, researchers increasingly adopt co-design methodologies to refine the already thought-out concept. Although this approach may initially seem appropriate, it collides with the core principles that underpin the value of design [18].

From a design perspective, one explores potential solutions through iterative experimentation and prototyping to assess their fit with the situation at hand, something Schön [64] called a “reflective conversation with the situation.” Problem and solution thus co-evolve [2], and testing prototypes is meant to learn, literally “a question embodied” [65]. In short, designers observe the current situation, formulate alternative ways forward, consider their likely impact, and test promising options to determine what works in practice [66]. Wartena and Kuipers [27] describe this as rapidness. If the problem definition is predetermined and the solution is fixed, it becomes impossible to learn from and respond to the unique needs of people in these contexts [67], as well as the constraints of the context itself (see Lesson 4). The unpredictability – which allows serendipity – in co-design is precisely what makes it transformative for human-centered innovation. The solution should therefore surface as the co-design team engages in collaborative learning through ‘rapid[ness]’ design and testing in situ [27].

Co-design dynamics are best characterised by intensity and uncertainty due to diverse perspectives and co-design’s emergent and generative nature [68]. Again, in mental health care, co-design involves working with individuals who may lack a design background, within teams of people with experiential, professional, and scientific knowledge, which ideally operate on equal terms in a unique context. Some co-design initiatives complement this process by providing training or capacity-building activities that help stakeholders understand the design process and collaborate more effectively across disciplinary boundaries [9]. These teams should collectively determine how to frame the problem, which concepts to develop into prototypes, and the criteria for testing those prototypes [18]. Rapid testing – while in tension with more rigorous research methodologies (see Lesson 1) – may reveal that a concept fails to address the problem adequately or that the problem itself requires reframing due to new insights gained during the process. Accordingly, both the problem and the solution must remain open to challenge and revision, as they co-develop throughout the process. The co-design team cannot fully anticipate discoveries in advance, and it is often through this iterative process of exploration and learning that ‘real’ innovation emerges.

### Lesson 3: Co-design is one approach among many to integrate stakeholder perspectives

Co-design is a valuable and established method for integrating stakeholder perspectives into mental health care innovation; however, it is not universally applicable. In contexts involving vulnerable or less verbally expressive populations, alternative design research approaches – often requiring additional methodological steps – can also facilitate the authentic inclusion of stakeholder perspectives [39]. Since the integration of lived experience and experiential knowledge is widely recognized as a key driver in the design of health interventions, particularly within mental health [9,10,69,70], co-design is frequently positioned as the preferred or ideal approach for achieving this integration. Nevertheless, the concept of “co-” in co-design presupposes a level of participation in which various stakeholders act as partners throughout the design process. In practice, this assumption does not always hold. Certain groups in mental health may be unable to participate as equal partners due to age, cognitive or functional limitations, language barriers, vulnerability, or contextual constraints. Similarly, in experience-based co-design in mental health care approaches are often adapted to accommodate trauma-informed, contextual, and population-specific conditions [71].

Nevertheless, Terlouw and Veldmeijer [39] indicate that design and innovation projects can achieve genuine stakeholder involvement even without full partnership. In fact, challenges surrounding participation and involvement should never be used as an excuse to exclude experiential perspectives. When individuals are less able to participate as equal contributors, several strategies exist to ensure their perspectives are integrated into the design process. These strategies often involve indirect elicitation of lived experience, iterative validation through testing, and expert interpretation to translate insights into design decisions. In these scenarios, the facilitator’s role becomes more complex, requiring additional methodological steps to surface, interpret, and validate stakeholder perspectives. This complexity is particularly apparent when direct involvement is limited or only feasible at later stages [39]. In such cases, perspectives can be accessed through proxies or informants and subsequently validated through structured testing with the target group. Reduced direct participation thus does not preclude relevant inclusion of experiential perspectives but requires a more deliberate and methodologically layered approach to ensure adequate representation.

Project leads should therefore carefully deliberate on methodological selection. If abductive design reasoning, iterative making/prototyping, artefact or service development, problem–solution co-evolution and explicit design decision-making is needed, co-design is a great choice. However, a broad range of adjacent approaches and traditions exists, each with distinct strengths and limitations relative to specific contexts and target groups. Not every project necessitates shared decision-making, and co-design should not be applied by default. Designers should maintain the capacity to reframe problems based on insights from both practice and theory. Project leads are encouraged to transparently justify how their chosen approach aligns with the characteristics of the target group and the specific design challenge.

### Lesson 4: Contextual constraints are indispensable inputs in the co-design process

Designers do not create solutions in isolation from the context in which they will be used, particularly in projects involving human behaviour. Instead, designers develop potential solutions in context and test these options early and often, enabling them to quickly learn about their match with the situation and the consequences they cause [64–66]. In mental health care, this context may for example include the therapy room when developing a therapeutic intervention, the digital systems clients use to access records and appointments, or broader system-oriented processes such as the pathway from referral to intake. These various domains share a common objective: addressing challenges by initiating purposeful change through the co-design of potential solution directions.

Even minor contextual constraints can require significant adjustments to framing, briefing, principles, and choices during the co-design of concepts, tailored to the specific situation. For instance, developing an innovative therapeutic intervention requires understanding the attitudes and needs of both professionals and patients, the implications for clinical practices, the demands placed on patients/clients, and the strategies for effective integration within the broader care system. Designing an intervention in isolation and subsequently implementing it is insufficient; the outcome must encapsulate experiential, professional, and scientific perspectives as a coherent narrative deployed within a unique ecosystem [18]. The value of potential solution directions should be assessed early in the co-design process by evaluating whether the artefact or design outcome resonates with the people it is designed for and fulfils its purpose within the relevant context. With this notion, involvement and participation in co-design are processes of collective learning and reflection to generate shared knowledge and understanding [72,73], embedded in broader institutional, organisational, and systemic structures, including funding mechanisms, research governance, disciplinary norms, and prevailing conceptions of evidence. Small-scale, context-specific qualitative tests are particularly valuable for revealing the underlying values of stakeholders in relation to the proposed solution or change.

## Domain 2: The collaborative knowledge production process

### Lesson 5: Align early on the epistemological paradigm through which knowledge is understood

Misunderstandings between experiential, professional, and scientific perspectives frequently arise because they hold different, often implicit, assumptions about what counts as valid knowledge and how it should be generated. Much of the mental health care literature is oriented toward effectiveness (rigour), a focus that aligns with the historical and scientific foundations of health research. The medical field has established a robust tradition of effectiveness studies across diverse health issues, which has driven significant disciplinary evolution and medical advancements. However, Blandford et al. [74] and Groeneveld et al. [75] highlight the challenges that emerge from cultural clashes in interdisciplinary collaboration, particularly between medical research and design research. Medical science and design culture differ fundamentally in their perspectives, approaches, and research methodologies. In mental health research, these epistemic imbalances are especially significant, as co-design can reinforce instead of transform existing hierarchies of power and knowledge when experiential knowledge is only recognized if it conforms to dominant scientific or professional reasoning, making co-practices ‘untenable’ and potentially harmful [76].

Research paradigms vary in their ontological assumptions (what reality is), epistemological criteria (what counts as knowledge), and methodological approaches (how knowledge is generated) to knowledge production [77]. Common paradigmatic lenses include for example (post-)positivism, social constructivism, pragmatism, and the participatory paradigm [78]. Much mental health research is conducted from a (post-)positivist perspective, which assumes a single reality characterized by order, patterns, and cause-and-effect relationships, where events are not random. Within this paradigm, the pursuit of truth is the primary value of research (rigour), and a variety of methodologies and methods may be employed to achieve this objective.

Concurrently, the mental health literature encompasses a wide array of studies employing co-design methods. While some of these – more medical focused – studies pursue ‘truth-finding,’ many – more design focused – studies are implicitly informed by pragmatic and participatory paradigms, where usefulness and practical impact for people in their contexts are prioritized (resonance and relevance). In such instances, research is expected to address real-world problems directly by intervening through making and testing. This divergence in paradigmatic orientation often results in misunderstandings, particularly in a field where the medical paradigm remains predominant. For example, designers may consider iterative prototyping and contextual testing as suitable methods for generating knowledge, while researchers operating within a (post-)positivist paradigm may primarily expect predefined hypotheses and standardized procedures. Epistemological differences like these frequently shape design decisions, methodological approaches, and the assessment of study quality. These disputes also reflect deeper differences in worldview. Firestone [79] accurately notes that “[t]here are, in fact, several reasons for selecting a methodological approach, but one’s decision often expresses values about what the world is like, how one ought to understand it, and what the most important threats to that understanding are.”

Paradigmatic differences are also evident in disciplinary orientations. Medical researchers typically focus on understanding and establishing current realities and practices, grounded in (post-)positivist assumptions. In contrast, designers aim to shape or represent new or improved practices through abductive reasoning. Consequently, these disciplines make distinct choices throughout the design and research process, including stakeholder involvement, the timing and nature of participation, and the role of evidence and knowledge. For medical researchers, evidence and theories serve as a central evaluative framework, whereas for designers, they provide guidance and a foundation for developing contextually impactful solutions [18]. Thus, greater attention to alternative paradigms and their interrelationship in co-design is advised – paradigms should be regarded as interconnected, each with a legitimate role in contributing to a more comprehensive understanding of mental health challenges and potential innovations.

### Lesson 6: Power relations need to be negotiated and reported from the outset

Co-design teams may collaborate through various methods and configurations. Meaningful co-design requires agreements regarding decision-making authority. Frequently, such agreements are absent and rarely documented in mental health studies [9]. Genuine participatory design calls for a shift from involving participants solely as informants to recognizing them as integral contributors to the design process [80]. Achieving meaningful co-creative inquiry, design, testing, and research – where each team member is acknowledged as an expert in their own knowledge and perspective [16] – demands shared power and transparent reporting of power dynamics [26]. Nevertheless, research indicates that the involvement of individuals with lived experience remains insufficiently reported [9,69,70,81]. Moreover, experiential perspectives are often adapted to fit prevailing hermeneutical frameworks and established research structures [82], and although their participation in decision-making is considered crucial, it is infrequently realized [26].

Such power sharing arrangements should be established prior to initiating a co-design project, not during or after the project. These arrangements may include jointly defining decision-making authority, clarifying who is responsible for different design decisions, and agreeing how disagreements will be negotiated and documented throughout the project. Power is not a binary attribute but it is shaped by multiple factors, including one’s social location, intersectional identity, and the specific context in which one operates. Epistemic pluralism in mental health [28], where each stakeholder maintains a degree of authority over their ways of knowing, is vital for co-design [18]. Innovation relies on the inclusion of divergent perspectives (see Lesson 1), which may not always align but should be valued, even when they generate friction. Examining these perspectives and the rationale behind them forms the foundation for productive collaboration, where dissensus serves as a guiding principle for establishing common ground [83]. Publicly documenting these discussions and clarifying decision-making processes are crucial for transparency regarding the collective development of ideas [18].

### Lesson 7: Maintain transparency about principles, choices, roles and responsibilities

A central reporting issue surfaces regarding the documentation of underlying design choices and principles, as well as the processes by which these are established and the roles and responsibilities of different stakeholders in these processes. As mentioned in Lessons 1 and 5, in much of the mental health literature, interventions are primarily described by their intended purposes and evaluated for effectiveness. Although outcome-focused reporting is valuable, it often obscures the design process itself, making it frequently unclear which design choices were made, how these decisions were informed by theory and practice, and why certain directions or perspectives were selected over others [9,27]. This pattern is also evident in a comprehensive review of behavioural design by Nielsen et al. [84], which found that theory-informed decision making was prioritized, while stakeholder-informed decision making was rarely reported.

The absence of design rationales and comprehensive descriptions of stakeholder involvement, and thus a lack of transparency in design reasoning, is particularly concerning in mental health care. To reiterate, design studies in this field focus prematurely on evaluating design outcomes – often after only the first iteration – to demonstrate average effects [9]. As a result, the reasoning underlying the artefact remains insufficiently understood. This reasoning is arguably the most critical aspect to make transparent, especially for co-design, as it is important in early stages to determine and understand whether the design outcome results from a deliberate, iterative design process and forms a coherent concept before large-scale testing and subsequent implementation. Instead of focusing primarily on design outcomes, it is recommended to document the decision-making process: which choices were made, how were they made and by whom, and how do they relate to existing knowledge and the principles upon which the design outcome is based [18]. Articulating this rationale enhances falsifiability and enables other researchers to understand, replicate, and extend previous design work, thereby strengthening cumulative and transferable knowledge development in mental health design.

Collectively, these seven lessons reveal a consistent and recurring pattern of design processes that are – explicitly or implicitly – guided by a combination of experiential, professional, and scientific stakeholder contributions, design principles, theories, and evolving design hypotheses, yet these elements are rarely reported in an integrated and transparent manner, including the roles and responsibilities of the participating stakeholders [9]. This lack of transparency in reporting needs attention, as it restricts the ability of others to learn from, reproduce or build upon the work. These reporting challenges motivate the need for a structured co-design rationale. The following section translates these lessons into a preliminary architecture that captures what was co-designed, how design decisions were reached, whose perspectives informed them, and how disagreements and responsibilities shaped the final outcome.

## The architecture of co-design rationales

**Fig 1:**
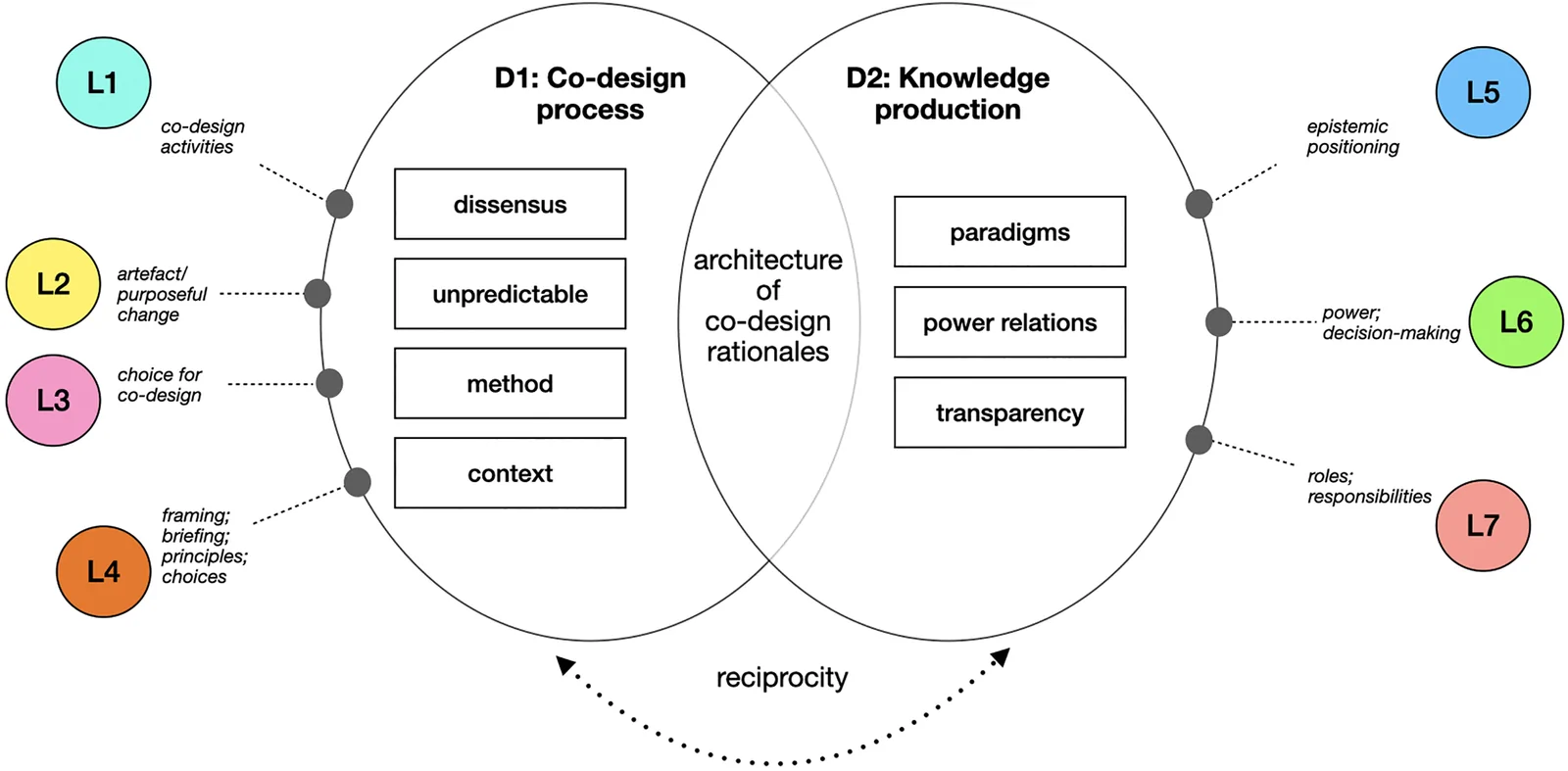
Overview of the seven lessons learned that informed the proposed architecture of co-design rationales. The lessons are organised into two interconnected domains: the co-design process and collaborative knowledge production. Together, these identify the elements that should be made transparent when reporting co-design in mental health care. Reciprocity is presented as the relational condition linking both domains. The arrangement of the figure is conceptual and does not imply a hierarchy or sequence of importance.

The first part of this article presented seven lessons learned concerning research paradigms, contextual constraints, divergent stakeholder perspectives, the continuum of participatory design approaches, serendipity and unpredictability as core mechanisms, power relations, and transparent reporting of participatory design and roles and responsibilities. We categorized these lessons for pragmatic reasons into two domains: the co-design process (D1) and the collaborative knowledge production process (D2). The present section establishes the foundation for ‘co-design rationales’ by transforming these into a preliminary architecture. See Fig 1 for a figural overview of the lessons learned and their key elements.

Section one established that co-design outcomes in mental health care consist of multiple components, tied to different ways of knowing, each serving a distinct function or value. Critical here is understanding the influence of these components to comprehend how the design outcome or change was achieved. To clarify this outcome, we introduce a simple heuristic equation, intended as a conceptual device, to articulate the value and focus of design (thus, for now, excluding the ‘co’ prefix):

A (*what; design outcome*) + B (*rationale; justification for design choices*) + C (*how; operational mechanisms of the design*) + D (*experience; user perception of the design*) = value of design

In sum, design research must describe (a) the design outcome, (b) its rationale, (c) its operational mechanisms, as well as relate these aspects to (d) the user experience within context. Design rationales are therefore a central pillar for understanding and explaining the potential success of a design outcome, evaluating context-dependent user experiences, and making explicit the design knowledge embedded in the outcome. This transparency enables other design researchers to learn from and build upon previous work, accounting for rigour, relevance, and resonance [27,85–88].

**Fig 2:**
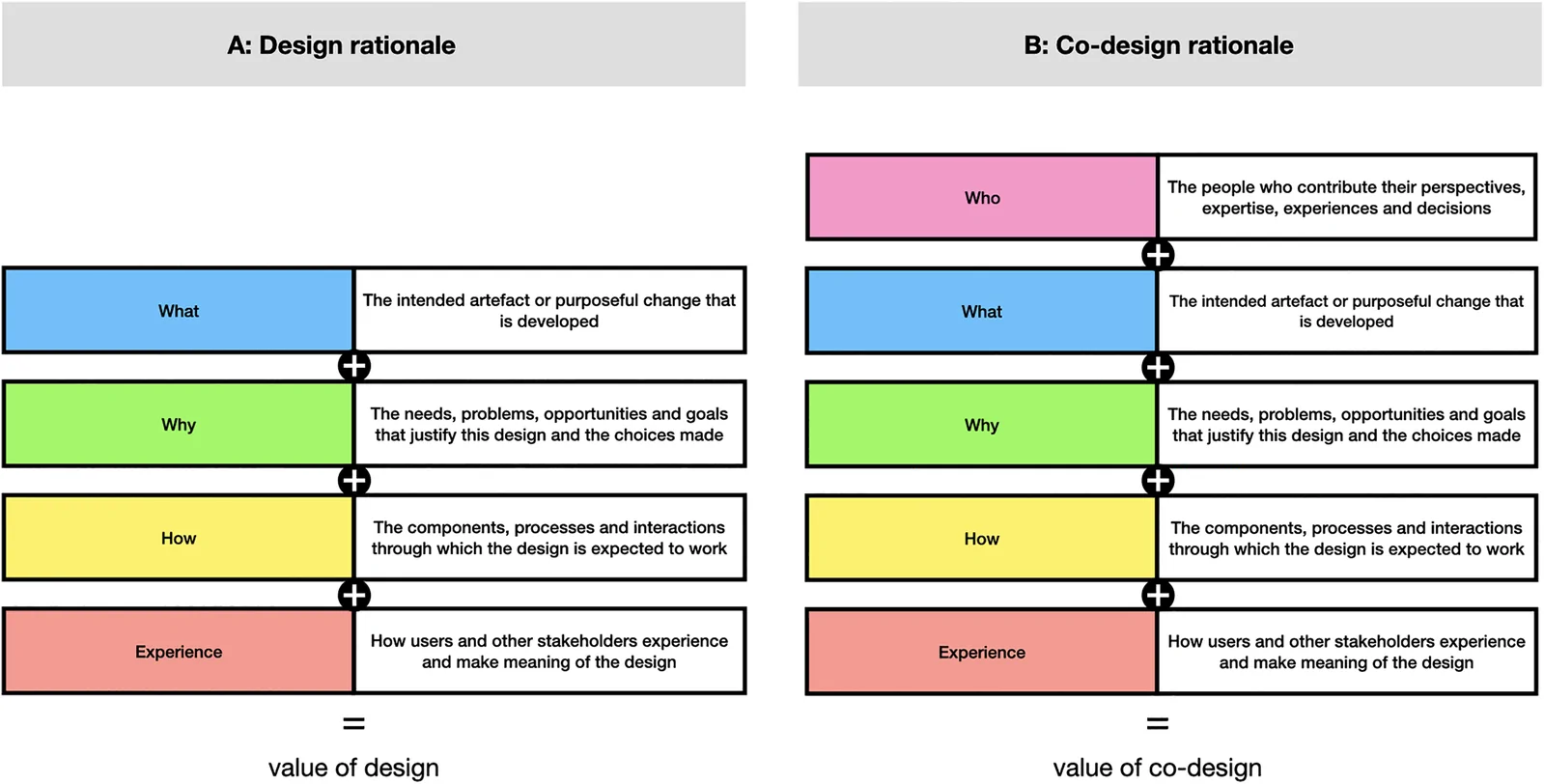
Comparison between a traditional design rationale and a co-design rationale. Whereas a design rationale documents the what, why, how, and experience of a design, a co-design rationale adds the who: the stakeholders whose experiential, professional, and scientific perspectives contributed to design decisions. The additional component makes collaborative knowledge production explicit and clarifies how stakeholder contributions shaped the resulting artefact or purposeful change.

For co-design, we argue throughout this essay that an additional factor must also be made explicit: the manner in which stakeholders with various perspectives and ways of knowing contributed to the process, and thus the outcome. We agree with Beresford [72] who pointed out that involvement and participation is about collective learning and reflection to generate shared knowledge and understanding. In the context of co-design, we refer to this as the encapsulation of shared and collective knowledge and learning, representing the “collaborative composition of design outcomes” [18]. In the current participatory context, where designers increasingly collaborate with stakeholders lacking formal design backgrounds and holding diverse perspectives, design rationales should therefore also document who contributed to each conceptual decision and how these decisions were reached. This aspect is often absent but, as demonstrated in the preceding lessons learned, essential here. The following heuristic equation further clarifies our argument. Co-design in mental health care can be conceptualized, in our view, as comprising the following interrelated factors:

A (*who; contributing stakeholders*) + B (*what; co-design outcome*) + C (*rationale; justification for design choices*) + D (*how; operational mechanisms of the design*) + E (*experience; user perception of the design*) = value of co-design (Fig 2)

The introduction of the ‘co-’ prefix adds a crucial dimension to ‘design’, ‘the who’ factor, which we prioritized as the first factor because it is central to the value of co-design: making transparent who contributed and in what manner to the co-design outcome. In essence, this addresses the origins of co-design as producing collaborative compositions. Although shared team-level processes of co-evolution in design require further investigation [89], we introduce the concept of the ‘co-design rationale’ here to clarify the roles and contributions of various stakeholder perspectives and team members (see Table 1).

**Table 1:** Key elements proposal for co-design rationales in mental health care co-design publications.

Domain 1: The co-design process	This domain focuses on the concrete design activities in co-design processes.	Example reporting questions
Design brief	Describe the design brief as formulated at the start of the project; specify who formulated it; explain on what insights this description was based.	What challenge was addressed? Who formulated the brief? Which assumptions guided the project?
Framing	Report whether the problem framing was considered provisional; describe how and by whom reframing was possible throughout the co-design process.	How was the framing understood? How did the framing evolve during the process?
Choice of co-design as design approach	Justify co-design as a deliberate choice; describe which type of design complexity or uncertainty legitimized this choice; discuss which other design or participatory approaches were considered and the reason why these were deemed unsuitable.	Why was co-design appropriate? Which alternatives were considered?
Co-design principles and choices	Formulate the co-design principles that guided the co-design process; describe how these principles were concretely translated into design activities and choices.	Which principles guided collaboration? How were they operationalised?
Co-design activities	Describe the concrete co-design activities used in the co-design process; report for each design activity the design problem it addressed, the type of knowledge production it was intended to support, the materials used, why this activity was considered appropriate under these conditions, what its goal was, and how the results relate to the goal.	Which activities were conducted? What design question did each address?
Theories	Describe the theories that supported the design and co-design process, which can for example be design theories, evidence-based theories in health or documented lived experience perspectives; report how these theories informed design choices; describe whether theory was used for explanation, inspiration, communication, or legitimation.	Which theories informed the design? How did theory influence decisions?
Design artefact/co-design end	Describe the result of the co-design process; explain how it resulted from design activities and collaborative work; discuss intended use, how it was tested, what contextual factors and constraints played a part; explain how the test results relate to its design rationale and intended use or purpose.	How are stakeholder perspectives visible in the final artefact?
**Domain 2: The collaborative knowledge production process**	**This domain addresses the ways in which stakeholders collaborate and contribute knowledge within co-design processes.**	**Example reporting questions**
Epistemic positioning	Specify which forms of knowledge were used as design input and report which knowledge was considered guiding for design choices; report which stakeholders were allowed to make abductive leaps; describe how all contributions were validated or rejected; report how paradigmatic tensions were considered.	Which epistemological assumptions informed the project? Were these discussed among stakeholders?
Roles and responsibilities	Describe who was involved, who held responsibility for which design choices and distinguish between contributions and decision-making power; report on the level of involvement of stakeholders and whether roles could shift during the design process.	Who participated? What was each stakeholder’s role? Could roles change over time?
Power	Describe institutional or organizational constraints that influenced design choices; report which design choices were open and not open to co-design and why.	Which decisions were open to co-design? Who made the final decisions and why?

**Fig 3:**
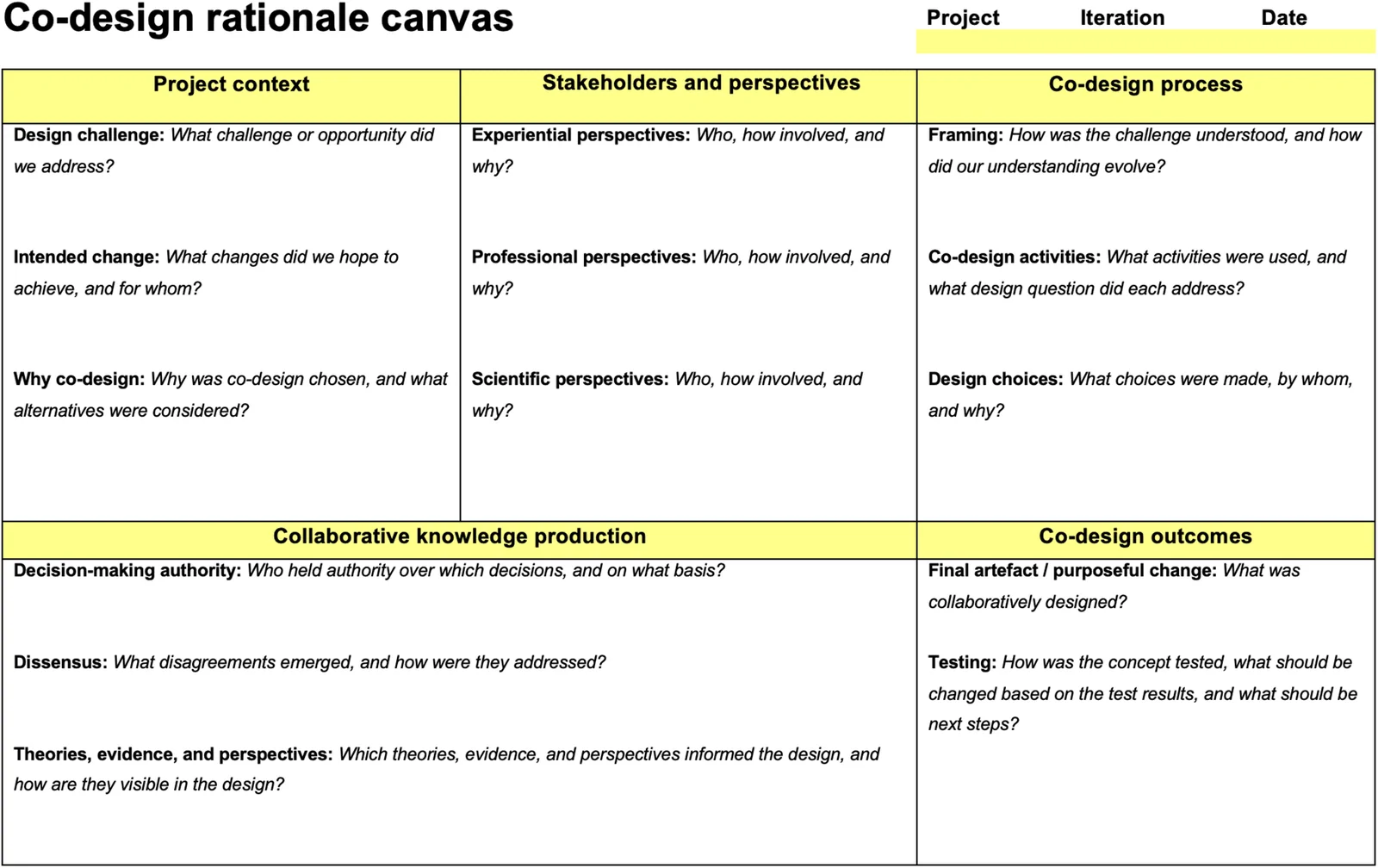
Version 1 of the proposed co-design rationale canvas. The canvas is intended as a living document that can be iteratively updated throughout a co-design project to document project context, stakeholders and perspectives, co-design process, collaborative knowledge production, and co-design outcomes. It may be used as an internal working document, to support manuscript reporting, or as supplementary material to enhance transparency and reproducibility. A fillable version of the canvas is provided in S1 File.

Drawing on extensive design rationale literature, the purpose of our proposal for implementing co-design rationale is to describe what was designed and to clarify why particular design choices were made, how these choices were informed by theory, practice, stakeholder contributions, and contextual constraints, and how they relate to the intended functioning of the design outcome in context [27,55,85,86,90]. Co-design rationales should be considered living documents, updated across iterations, discussed with stakeholders, and used to support shared decision-making and to report collaborative design reasoning in design and research studies (Fig 3).

Co-design rationales are intended to be flexible. They may function as internal working documents throughout a project, inform the reporting within a manuscript, or be shared as supplementary material to enhance transparency where appropriate. A significant consideration, however, is the potential practical burden involved in maintaining living co-design rationales. Although these rationales enhance transparency in reporting collaborative design processes, they may also increase documentation requirements and unintentionally reinforce administrative or academic control. Future research should investigate how co-design rationales can balance transparency with feasibility, while minimizing undue burdens on lived experience and community partners.

We will illustrate in short what co-design rationales can contribute by using two cases that focused on designing with people with lived experience of psychosis, professionals, and researchers from our own research group. The first case is a cautionary tale, while the second illustrates a potential best practice of what co-design rationales could contribute.

### Two illustrative cases: Bias Blaster and In Picture Approach

The first case examines Bias Blaster [91], a project that sought to develop a serious game to address implicit bias. While a range of stakeholders participated throughout the design process, key decisions – including the framing of the challenge and the solution direction – were primarily influenced by scientific and professional perspectives. Experiential perspectives contributed mainly to aesthetic elements, such as colour schemes and game features, whereas alternative suggestions were dismissed for lacking sufficient evidence. As a result, opportunities for reciprocity and reframing were limited, and stakeholder influence, in particular the impact of experiential perspectives on decision-making, was uneven. These dynamics ultimately shaped the final artefact, which reflected the initial framing as opposed to a co-evolved, collaboratively developed alternative. In the absence of a documented co-design rationale, it remains unclear how stakeholder input affected specific design choices, which alternatives were considered or rejected, and how power relations influenced the artefact. *Bias Blaster* thus demonstrates that the lack of a co-design rationale can obscure collaborative design reasoning, even in projects with significant stakeholder participation.

The second case, conducted more than ten years later, concerns the *In Picture Approach* [55]. This project aspired to co-design a method that enables individuals experiencing mental distress to represent their experiences in ways that meet their specific needs. Design choices were developed through iterative collaboration among individuals with lived experience, professionals, and researchers, with each perspective – experiential, professional, and scientific – jointly informing the evolving artefact. While stakeholders did not always reach consensus, they consistently engaged with tensions and negotiated decisions, which fostered reciprocity throughout the design process. These collaborative interactions produced a booklet and method that reflected context-specific choices and facilitated collective learning. The resulting publication documents much of the underlying design reasoning by making stakeholder contributions, design decisions, design principles, and theoretical considerations, such as the supporting and working theories, explicit. Although epistemic positioning and paradigmatic assumptions remain implicit, this case highlights the value of documenting collaborative design reasoning and demonstrates the potential contribution of co-design rationales to transparent and accountable co-design practice.

### Reciprocity as ‘sine qua non’ for co-design in mental health care

In addition to the need for co-design rationales in mental health care innovation the seven lessons also show that co-design is fundamentally concerned with the quality of relationships through which design decisions are shaped. We are, however, not the first to postulate that reciprocity and participation in design processes go hand-in-hand. For example, Dreessen et al. [92] define reciprocity in participatory design as “[..] a mutual exchange, which can entail a direct gain for the participant or a form of reciprocity in which each participant acts with the interest of the other in mind.”

**Fig 4:**
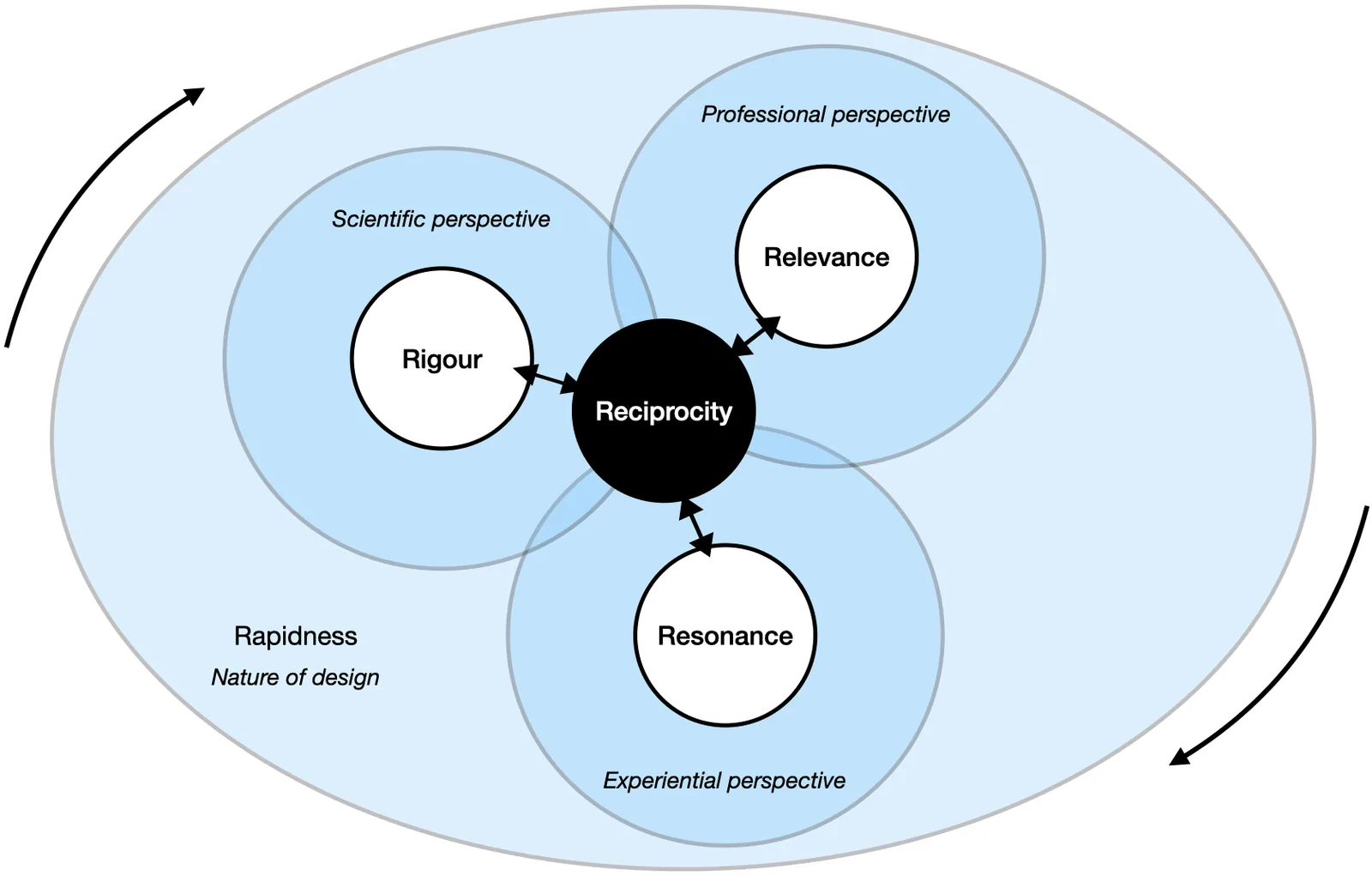
Reciprocity as the relational condition that is necessary for meaningful co-design. Reciprocal co-design is characterised by the mutual influence of experiential, professional, and scientific perspectives throughout framing, prototyping, testing, and decision-making. Reciprocity makes differences in perspectives visible and negotiable, enabling collaborative learning and the co-evolution of design decisions across iterative design cycles.

We expand on this definition by arguing that co-design processes depend on the ongoing exchange of dissenting perspectives, experiences, interpretations, and expertise among stakeholders from experiential, professional, and scientific backgrounds. Researchers, designers, clinicians, managers, service users, caregivers, individuals with lived experience, and so on, do not by default participate in co-design processes with the same goals, values, and worldviews. Accordingly, the collaborative production of design knowledge relies on relational and social conditions such as reciprocity that encourage such knowledge exchange through perspective-making and -taking and promote mutual learning [57,93]. From this perspective, reciprocity in co-design in mental health care refers to the mutual recognition of diverse stakeholders as legitimate contributors to the co-design process by making design choices and the ‘gameness’ to both influence and be influenced by others. This bidirectional exchange allows participating stakeholders to contribute their expertise while remaining open to having their own viewpoints, assumptions, interpretations, and priorities reshaped through interaction and discussion (Fig 4).

Within a co-design rationale, reciprocity is evident in the documented evolution of stakeholders’ ideas, framing, design choices, prototyping, and testing throughout iterative design cycles. In reciprocal co-design processes, perspectives influence each other over time and co-evolve, which demonstrates openness to mutual learning and adaptation among participants. Correspondingly, mental health co-production studies also indicate that reciprocity relies on relational and emotional mechanisms, such as establishing safe spaces, facilitating decision-making, encouraging shared reflection, fostering vulnerability, and continuously renegotiating power dynamics as opposed to presuming that power can be equalized at the outset of a project [94,95]. Importantly, reciprocity should not be interpreted as eliminating existing power asymmetries but as creating conditions in which power relations become explicit, open to reflection, and negotiable throughout the co-design process. Ongoing critical reflective practice and dialogue are known to be essential to facilitate equal relational processes in co-design [96]. Unlike rigour, relevance, and resonance [27], reciprocity does not primarily characterize a qualitatively distinct perspective of a participating stakeholder in the co-design process but should be regarded as the hallmark that enables the co-learning between those ways of knowing.

Let us provide an example of why reciprocity is most important by illustrating a few common tensions one can encounter in co-design processes. Imagine a co-design project with diverse stakeholders in mental health care. An excessive emphasis on rigour in the co-design process may shift the objective toward producing generalizable knowledge or truth finding, potentially overlooking important nuances as required for innovation. This also creates tension with resonance, as the emphasis on rigour risks promoting scientific and professional knowledge over experiential knowledge. On the other hand, an uncritical overemphasis on resonance can deliver novel and unique first-person perspectives yet may also result in misleading representations of specific groups, while at the same time pushing professional and scientific perspectives to the background. Similarly, a predominant focus on relevance can lead to adequate considerations of pragmatic and contextual goals and needs for professional purposes, however, established scientific knowledge and first-person perspectives may as a result be set aside. This all takes place within a paradigm that prioritizes rapidness, as an inherent methodological feature of design, which promotes swift iteration and development in the co-design process, but may compromise thoroughness on the research end from a (post-)positivist stance.

Co-design processes always involve such tensions [73], which all result from valid and legitimate perspectives. These tensions thus need to be productively explored at their deepest core, as the clash and exploration of tensions is exactly where innovation occurs. As Palmer et al. [97] argued, co-creating knowledge requires respectful dialogue, navigating differences, and – what we perceive as most crucial – working through such disagreements, which is precisely why reciprocity is a necessity.

In sum, reciprocity can be understood as the necessary relational mechanism, or hallmark, through which the co-design process enables mutual learning as well as generating new knowledge by influencing and being influenced, even if this is uncomfortable for the stakeholders. It establishes the conditions required for epistemic pluralism by enabling stakeholders to move beyond consultation toward productive exchange of insights and perspectives in a delicate balancing act, without pursuing consensus or prioritizing one perspective or activity system. To quote Freire [98]: “Knowledge emerges only through invention and re-invention, through the restless, impatient, continuing, hopeful inquiry human beings pursue in the world, with the world, and with each other.”

Thus, reciprocity in co-design extends beyond mere participation or presence; it is *‘sine qua non’* (a necessary condition without which something, i.e., co-design, is not possible), and it requires that all perspectives have the capacity to influence collective understanding and subsequent design decisions. Again, as argued throughout this section, co-design rationales can make reciprocity visible by documenting how stakeholders influenced one another throughout the process, how disagreements were negotiated, how experiential, professional, and scientific perspectives shaped design decisions, and whether opportunities for mutual and collective learning were created.

## Concluding remarks

The central contribution of this essay is the introduction of co-design rationales as living documents that make collaborative design reasoning transparent and highlighting reciprocity as necessary for co-design. Throughout this essay we have argued that co-design rationales provide a structured way to report how experiential, professional, and scientific perspectives shaped framing, design decisions, and the resulting artefact or purposeful change in a reciprocal way. However, in our view, these suggestions also require several important reflections and remarks, which we aim to address in this section.

Co-designers in mental health care must recognize that co-design projects require shared ownership among stakeholders with diverse experiential, professional, and scientific backgrounds, promote trust and build relationships between them to arrive at their collaborative objective [99]. One notable contribution that potentially supports this is the Empathic Co-Design Canvas [100]. By analysing case studies, Smeenk [100] expanded on the Design Choices Framework introduced by Lee et al. [101]. The resulting canvas promotes discussion about challenges such as stakeholder involvement, purpose of change, co-design focus, intended impact, activities, and results, enabling all stakeholders to negotiate the conditions under which co-design projects occur, to support more deliberate and transparent collaboration. We highlight this canvas because it reveals a layer of co-design that is also central to our essay: the terms under which collaboration with diverse stakeholders takes place. Our proposal for using co-design rationales therefore is compatible with the framework by Lee [101] and canvas by Smeenk [100], as they all tend to shift the focus from co-design as a collection of participatory activities to co-design as a deliberately organised and negotiated process that embraces distinct types of knowledge. Co-design rationales also expand on this by reporting dissenting stakeholder perspectives and connecting these with the decisions made.

Another reflection is our specific – and deliberate artificial – demarcation between experiential, professional, and scientific perspectives we used throughout this essay. Practice is, of course, far more complex, where various ways of knowing are far more intertwined than our categorization suggests. Researchers, for example, sometimes hold a dual position as researchers with lived experience [102] and professionals as professionals with lived experience [103]. At the same time there are also people with lived experience that work as experts by experience and are researchers in academia. Moreover, the first author (LV) is a researcher with lived experience in mental health care. However, our categorization is chosen for clarity, to illustrate how different, in the words of Star and Griesemer [53], “activity systems” can collide in co-design. By using these terms, we tried to make these systems transparent, allowing future projects to work mindfully with these challenges and tensions toward a productive exchange of perspectives.

Further, project leads should consider how teams transition between ideas in an iterative and non-linear manner. For those aiming to build upon existing concepts or evaluate products within specific contexts, the co-design rationale offers critical awareness of the development of design choices through collaboration and co-learning, as well as the influence of differing perspectives [18]. The practical relevance of co-design rationales in mental health care is readily apparent. For example, the ongoing redesign of the *Diagnostic Statistical Manual of Mental Disorders* (DSM) has stimulated extensive debate about how people with lived experience should be meaningfully involved in shaping future diagnostic systems [104,105]. Recent correspondence has argued that, while lived experience involvement is increasingly recognized as important, greater transparency is needed regarding how experiential perspectives influence design decisions and epistemic governance throughout the redesign process [106,107]. In response, the DSM-taskforce acknowledged the need for appropriate strategies that enable meaningful involvement of people with lived experience [108]. Co-design rationales could provide one such strategy by making design reasoning, epistemic contributions, negotiations, and decision-making processes more explicit and transparent.

With this notion, co-design rationales can provide a potentially transferable account of collaborative design reasoning that may inform future projects in similar or distinct settings. This approach is consistent with complex intervention guidance, which treats development, theory, context, implementation, and evaluation as interdependent elements [109]. Additionally, co-design rationales can clarify why particular design outcomes resonate with specific end-users in specific contexts [27]. Nevertheless, while the introduction of co-design rationales represents an initial step, it remains insufficient to fully capture the complex and temporal dynamics of co-design processes, as critical factors such as categories, narratives, and value tensions often develop through interactions that conventional documentation methods may not adequately capture [110]. Although we contend that co-design rationales present a promising method for enhancing the transparency of collaborative design reasoning, their practical implementation necessitates empirical investigation. Subsequent research should assess the feasibility, acceptability, and utility of these rationales in various co-design contexts, and determine whether their use solely increases transparency or also facilitates more reflective and reciprocal decision-making throughout the design process.

As a fifth reflection, we wish to highlight the challenges one can encounter when aiming for genuine co-design in mental health care, whether using co-design rationales or not. As Veldmeijer [18] argued, the current power asymmetries in mental health care and the iterative nature of design make it difficult for a designer to engage in reflection and share decision-making with various participating stakeholders. In these design activities, designers must navigate the tension between preserving authentic voices and avoiding the reinforcement of stigma, which requires a genuinely safe and inclusive space that is not always available in mental health care organizations. This is compounded when one perspective carries more influence than the other, especially when experiential perspectives depart from professional and scientific perspectives. Since professional and scientific perspectives are given more weight than first-person perspectives in mental health care, co-design activities are easily constrained. As a result, it can be tempting – which is problematic – to replace iterative and rapid design with a linear and predictable process in which the design outcome is fixed from the outset and important stakeholders are only consulted to refine its already developed concept.

On a final note, we reiterate that all perspectives, whether experiential, professional, or scientific, are relevant for transformation in mental health care, and that we have to productively work together with and through these tensions and disagreements to allow innovation to ‘emerge’. To move forward, we, together, must make it willingly (un)comfortable [18]. When claiming and reporting co-design, it is thus essential to specify who influenced particular design decisions, the perspectives involved, the basis of authority, and the impact on the resulting artefact or purposeful change.

## Conclusions

In conclusion, co-design in mental health care involves collaboratively creating human-centred outcomes with stakeholders who possess experiential, professional, and scientific expertise, while ensuring reciprocity and transparency in design processes. However, when the rationale behind design choices is not reported, the knowledge embedded within design outcomes remains concealed, thereby limiting insight into design activities. Co-design rationales, as introduced in this essay, enables researchers who use co-design to elucidate how collaborative decisions clarify the intended use of design outcomes and inform user experiences within specific contexts. Documenting both successful and unsuccessful outcomes through co-design rationales yields practice-based insights that advance understanding of the emergence and adaptation of mental health innovations and purposeful change across diverse settings. Without co-design rationales, we may know that stakeholders participated, but not how their perspectives shaped the outcome or where reciprocity broke down.

## Supporting information

S1 File Fillable co-design rationale canvas (Version 1). A fillable Microsoft Word template of the proposed co-design rationale canvas, designed to support the iterative documentation of design decisions, stakeholder contributions, and collaborative knowledge production throughout the co-design process.
(DOCX)
