# Supplementary material for: Co-design rationales as living documents for reporting the exchange of experiential, professional and scientific perspectives in mental health care innovation
Source: PLOS Ment Health. 2026 Aug 20;3(8):e0000689. doi: 10.1371/journal.pmen.0000689 (PMC13492791; doi:10.1371/journal.pmen.0000689)
Supplement: Supplementary file 1 [file pmen.0000689.s001.docx]

| Project | | Iteration | | Date |
| --- | --- | --- | --- | --- |
|  |  | |  | |

**Co-design rationale canvas**

| **Project context** | **Stakeholders and perspectives** | **Co-design process** |
| --- | --- | --- |
| **Design challenge:** *What challenge or opportunity did we address?*  **Intended change:** *What changes did we hope to achieve, and for whom?*  **Why co-design:** *Why was co-design chosen, and what alternatives were considered?* | **Experiential perspectives:** *Who, how involved, and why?*  **Professional perspectives:** *Who, how involved, and why?*   **Scientific perspectives:** *Who, how involved, and why?* | **Framing:** *How was the challenge understood, and how did our understanding evolve?*  **Co-design activities:** *What activities were used, and what design question did each address?*  **Design choices:** *What choices were made, by whom, and why?* |
| **Collaborative knowledge production** | | **Co-design outcomes** |
| **Decision-making authority:** *Who held authority over which decisions, and on what basis?*  **Dissensus:** *What disagreements emerged, and how were they addressed?*  **Theories, evidence, and perspectives:** *Which theories, evidence, and perspectives informed the design, and how are they visible in the design?* | | **Final artefact / purposeful change:** *What was collaboratively designed?*  **Testing:** *How was the concept tested, what should be changed based on the test results, and what should be next steps?* |
